# A Linear Combination of Pharmacophore Hypotheses as a New Tool in Search of New Active Compounds – An Application for 5-HT_1A_ Receptor Ligands

**DOI:** 10.1371/journal.pone.0084510

**Published:** 2013-12-18

**Authors:** Dawid Warszycki, Stefan Mordalski, Kurt Kristiansen, Rafał Kafel, Ingebrigt Sylte, Zdzisław Chilmonczyk, Andrzej J. Bojarski

**Affiliations:** 1 Medicinal Chemistry Department, Institute of Pharmacology, Polish Academy of Sciences, Kraków, Poland; 2 Medicinal Pharmacology and Toxicology, Department of Medicinal Biology, Faculty of Health Sciences, UiT The Arctic University of Norway, Tromsø, Norway; 3 Department of Cell Biology, National Medicines Institute, Warsaw, Poland; University of Bologna & Italian Institute of Technology, Italy

## Abstract

This study explores a new approach to pharmacophore screening involving the use of an optimized linear combination of models instead of a single hypothesis. The implementation and evaluation of the developed methodology are performed for a complete known chemical space of 5-HT_1A_R ligands (3616 active compounds with *K*_i_ < 100 nM) acquired from the ChEMBL database. Clusters generated from three different methods were the basis for the individual pharmacophore hypotheses, which were assembled into optimal combinations to maximize the different coefficients, namely, MCC, accuracy and recall, to measure the screening performance. Various factors that influence filtering efficiency, including clustering methods, the composition of test sets (random, the most diverse and cluster population-dependent) and hit mode (the compound must fit at least one or two models from a final combination) were investigated. This method outmatched both single hypothesis and random linear combination approaches.

## Introduction

A pharmacophore model (also called a pharmacophore hypothesis) is one of the most important concepts in medicinal chemistry. It is defined as the spatial orientation of different features of a molecule (thus, pharmacophore modeling is a ligand-based method) required for the activity towards a biomolecular target [1–3]. Such a model can be used to describe a large number of structurally diverse compounds with only a handful of general features. Pharmacophore filtering is widely used in virtual screening campaigns [4–10] and in other drug development processes [11,12] This filter may be applied as a standalone [7,8] or as one of the subsequent steps in a screening cascade [9,10].

The attempts at pharmacophore modeling the known ligands of 5-HT_1A_R [13–27], a well-recognized therapeutic target [28,29] also intensively studied in our laboratory [30–33], have focused solely on visualizations and explanations in SAR studies [13–27]. Only very recently published pharmacophores of 5-HT_1A_R ligands were intended for use in virtual screening (VS), however only for the off-target activity of α1-adrenoceptor antagonists [34]. 

It is nearly impossible to define a universal model that covers the entire chemical space of the ligands of a particular target. The use of multiple models at once led to search parameter improvements, yet the arbitrariness of model selection makes it strongly dependent on the researcher’s knowledge and experience. To address the downsides of pharmacophore screening, we developed a novel approach involving the use of a carefully selected collection of pharmacophore models instead of a single hypothesis. The primary goal of the research was to develop and to evaluate a screening protocol that utilized a linear combination of pharmacophore models, i.e. a collection of individual hypotheses covering as much as possible the chemical space defined by ligands of a particular target. From the single hypotheses, created from ligand clusters, a group of models with the best combined performance (chosen using a homemade script) was selected and evaluated on various test sets. In addition, the proposed best combinations were compared with a single hypothesis and with randomly composed groups of pharmacophore models of equal length. 

## Materials and Methods

### General

**Figure 1:**
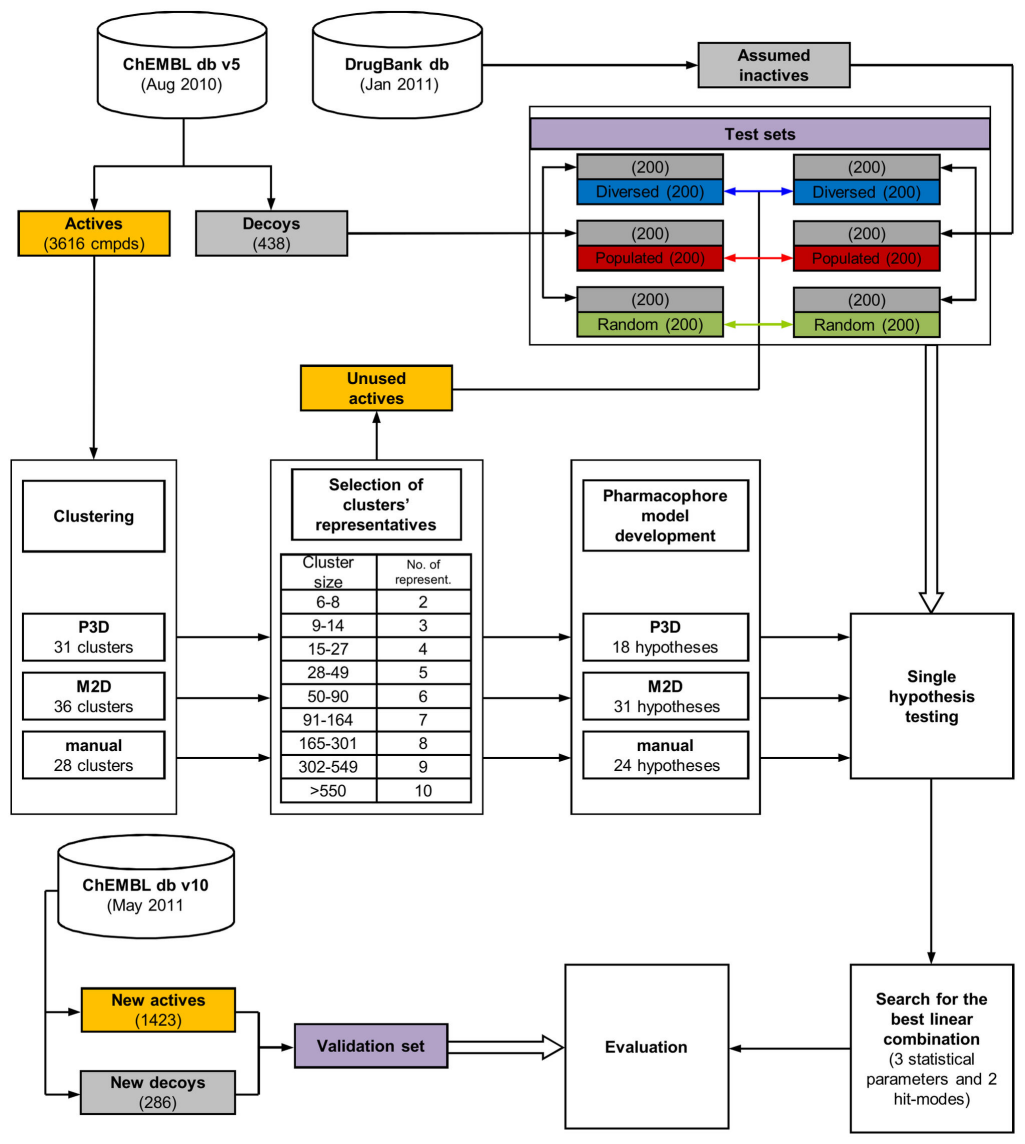
The development of an optimal combination of pharmacophore models. The development of an optimal combination of pharmacophore models. Transparent boxes show the logical steps of the workflow; cylinders represent data sources; colored boxes reflect the compound character: gray – inactives, orange – actives or the active’s selection method (blue, red or green), which is consequently used in subsequent figures. The population of the compound set is given in brackets. Thick arrows indicate the use of data sets.

Figure 1 shows a protocol applied for the development of the optimal linear combination of the pharmacophore models. Compounds with proven activity toward 5-HT_1A_R were acquired from the ChEMBL database version 5 [35], clustered, and structures representative of each cluster were used for the construction of a pharmacophore hypothesis. Each model underwent evaluation via test sets composed of active compounds, true decoys and ligands retrieved from the DrugBank [36] that were assumed inactive. The best linear combinations of pharmacophore models were then composed and validated with new compounds (both active and inactives) retrieved from the ChEMBL database v10 [37].

### Data sets

**Figure 2:**
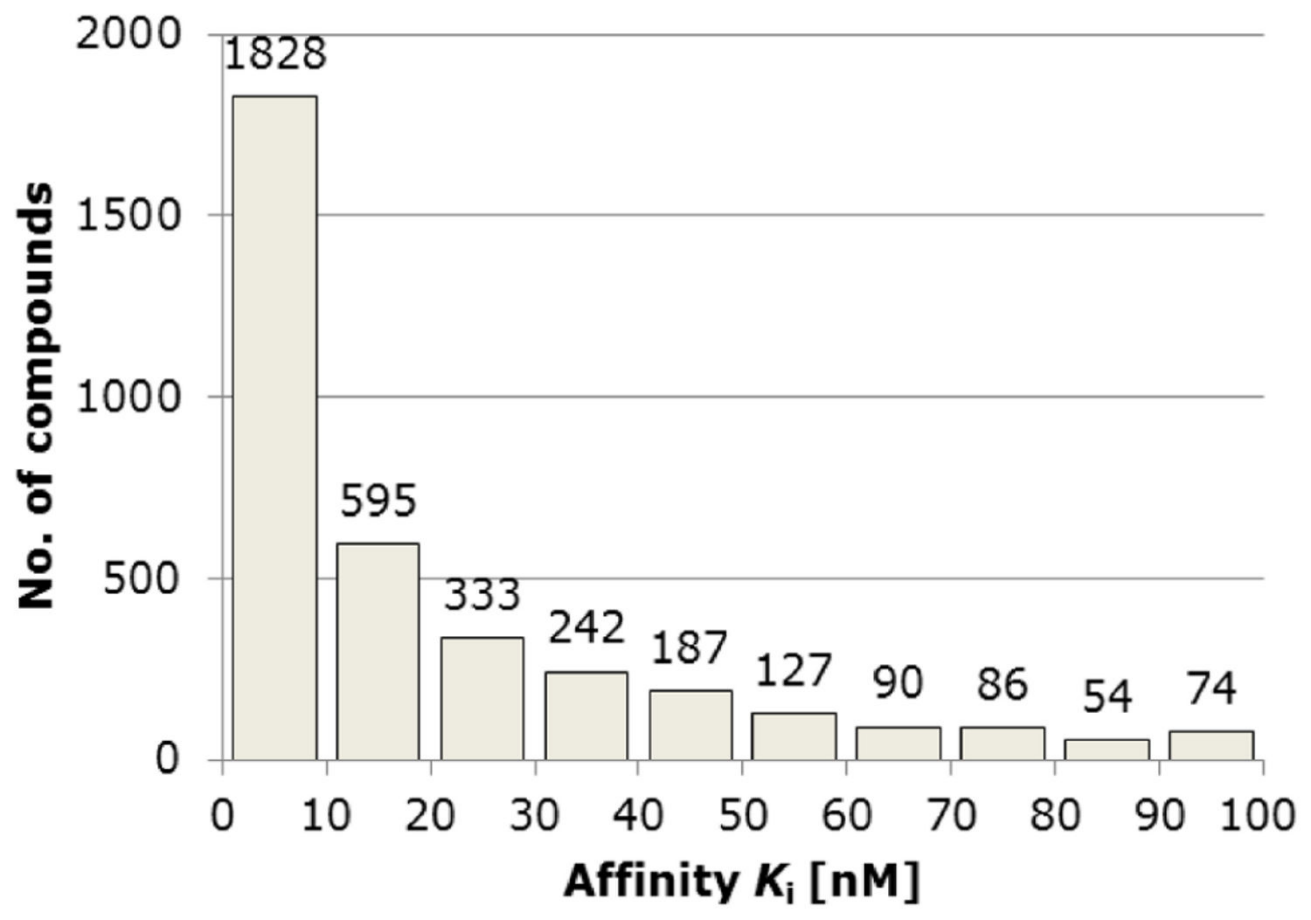
Affinity distribution of 3616 5-HT_1A_R ligands retrieved from the ChEMBL database version 5. Affinity distribution of 3616 5-HT_1A_R ligands retrieved from the ChEMBL database version 5.

The source of the active compounds was the ChEMBL database version 5 (August 2010), containing 5-HT_1A_R ligands retrieved from approximately 520 published papers. Due to a large diversity of activity measures, only the compounds with defined *K*_i_ (IC_50_ – assumed as 2×*K*_i_, p*K*_i_ or pIC_50_ were converted to *K*_i_) as assayed on human cloned receptors or on rat cloned or native receptors, were taken into account. In the case of multiple data for one ligand, the *K*_i_ and human receptors were given preference; a median value was used in the case of many biological results. The ligands were defined as active when their binding constant was lower than or equal to 100 nM; the threshold of inactivity was set at 1000 nM. The resulting sets consisted of 3616 active (more than half with *K*_i_ values lower than 10 nM – see Figure 2 for details) and 438 inactive (decoy) compounds (Figure 1).

### Clustering

Three methods of clustering were applied: 3D pharmacophore fingerprint-based (P3D), MOLPRINT 2D fingerprint [38]-based (M2D) and the manual – classical method (grouping the compounds by a common core). The P3D and M2D approaches were performed using the Hierarchical Clustering feature in Canvas [39].

For the P3D method, 31 clusters were created using the Kelly criterion [40]. After merging the singleton and doubleton subsets into a special class, 28 clusters containing 8–497 compounds were obtained.

The same approach, applied to MOLPRINT 2D fingerprints, left one cluster considerably larger than the rest, and its recurrent splitting (applied four times) resulted in a total of 36 collections consisting of 6 to 744 compounds each.

**Figure 3:**
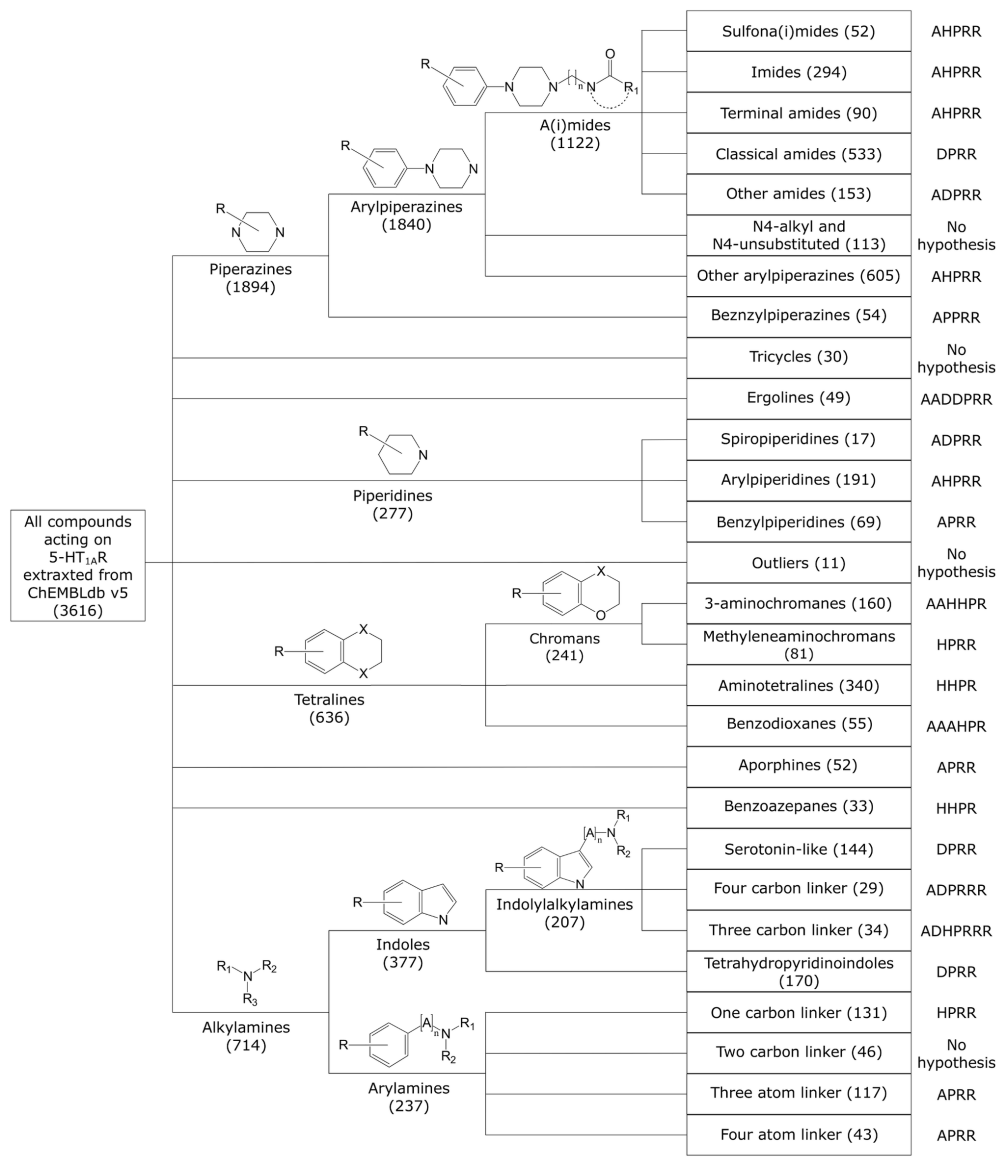
A dendrogram obtained using the manual clustering procedure. A dendrogram obtained using the manual clustering procedure. The number of compounds comprising each cluster is given in brackets. The last column presents a feature composition of the pharmacophore model created for a given cluster. The feature abbreviations used are: hydrogen bond acceptor – **A**, hydrogen bond donor – **D**, hydrophobic group – **H**, positively charged group – **P**, aromatic ring – **R**.

The manual clustering generally followed the classification of 5-HT_1A_R ligands described in the literature (9 basic classes) [41–43]. However, more subgroups were then created, e.g. for arylpiperazines [44] (Figure 3). In the case of the alkylamines (714 compounds), indole derivatives were first extracted and, with the exception of the tetrahydropyridoindoles, were divided depending on the distance between two crucial pharmacophore features: an aromatic system and a basic nitrogen atom. The entire procedure developed 28 clusters, each containing 17 to 605 compounds (Figure 3). 

### Pharmacophore model development

Cluster representatives, in number proportional to the cluster’s size (Figure 1), were selected using the diversity-based selection tool in Canvas (similarity metric – Soergel distance; compounds selection algorithm – sphere exclusion; sphere size – 0.5; initialization – random with random seed). The selected representatives were further used as a basis for the development of a pharmacophore model using Phase [45] under default settings (conformers generated during search, 10 conformers per rotatable bond; not more than 100 conformers per structure; relative energy window between conformations – 10 kcal/mol; RMSD tolerance for match – 2 Å). The best hypothesis for each ligand class must have mapped at least half of the input compounds. Among these hypotheses, the one with the maximum number of features, the highest matching rate and the best selectivity score was selected for further research. For some of the clusters, none of the hypotheses met all the requirements (13 for P3D, 5 for M2D and 4 for manual grouping).

### Test sets

**Figure 4:**
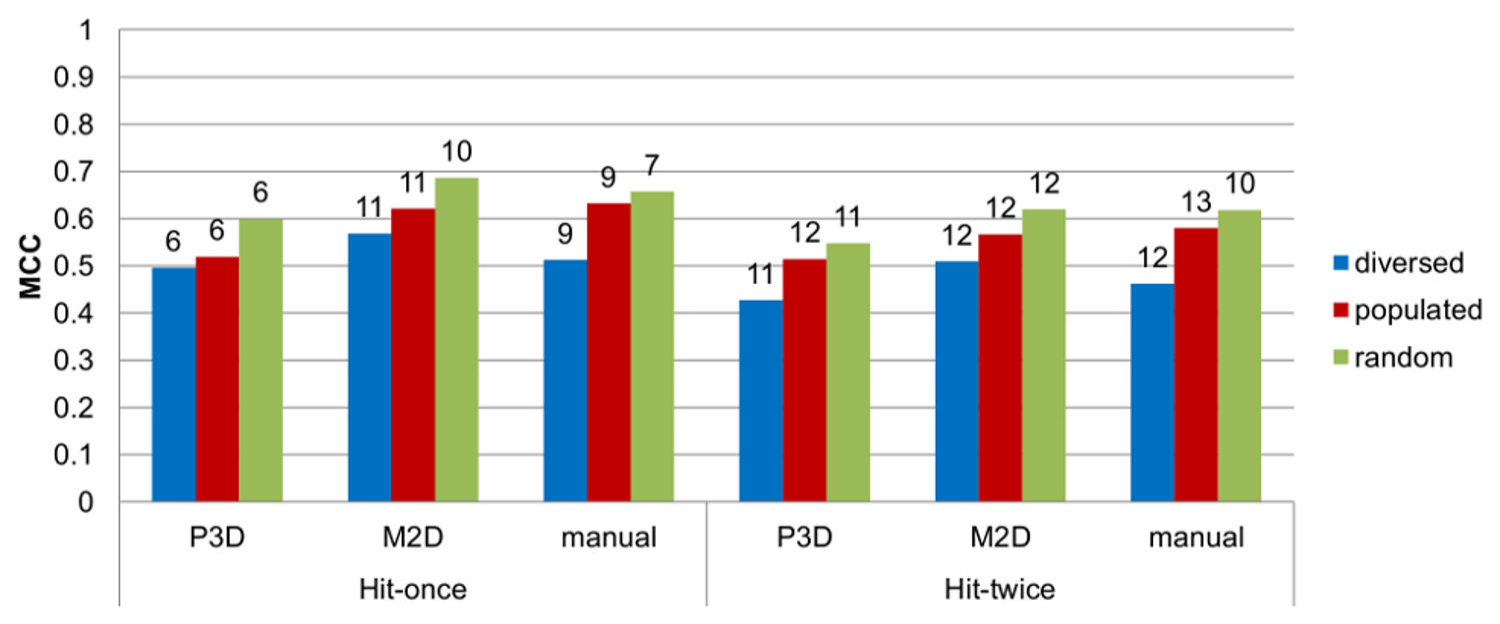
The optimized values of MCC for each possible scheme. The optimized values of MCC for each possible scheme. The length of combination is shown at the top of the bars. The composition of combinations based on the manual clustering approach is shown in Figure 5.

**Figure 5:**
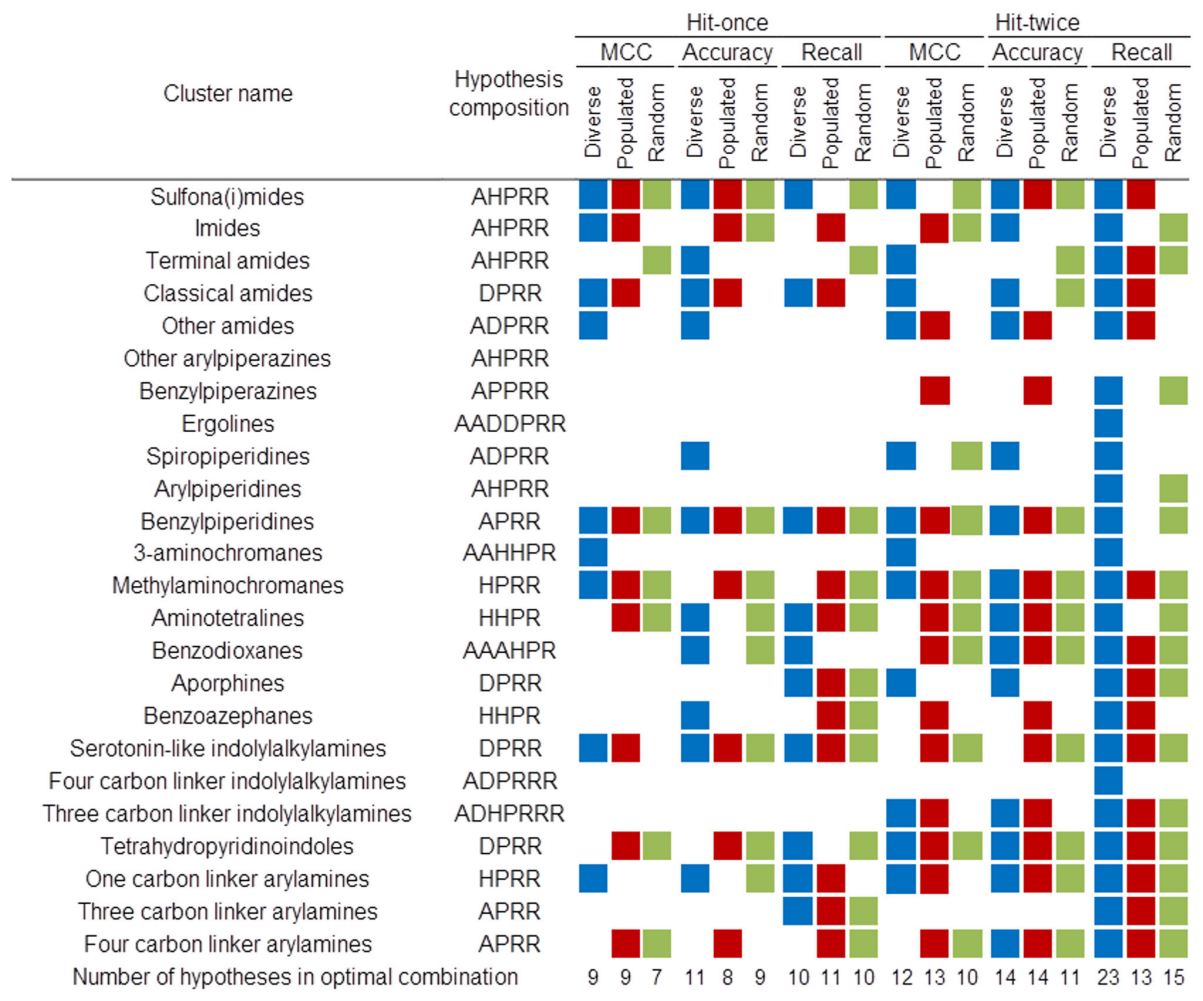
A composition of each top-ranked linear combination obtained using the manual clustering procedure. A composition of each top-ranked linear combination obtained using the manual clustering procedure. Each filled square denotes presence of a hypothesis developed on a particular cluster in the optimal combination for appropriate conditions. Colors code the type of the test set: blue – diverse, red – populated, and green – random. The last row contains the total number of hypotheses forming a respective top-ranked combination. The values of the optimized statistical parameters for manual clustering are shown in Figure 4, and those for accuracy and recall are shown in Figures S1 and S2, respectively. The exemplary linear combination (manual/random/hit-once; 7 hypotheses long) is shown in Figure 9.

**Figure 6:**
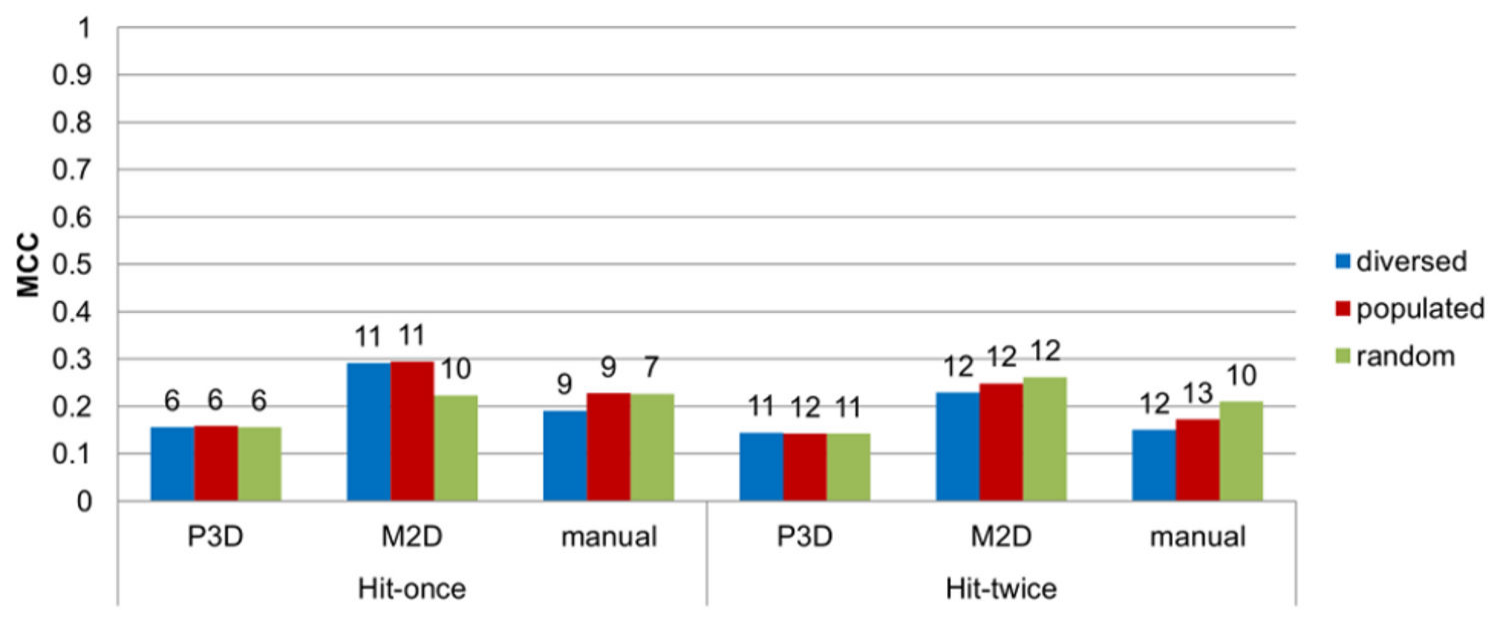
The MCC results for the validation of the top linear combinations. The MCC results for the validation of the top linear combinations. The length of combination is shown on top of the bars.

All obtained models were first tested on three pairs of 400-compound sets that were composed of an equal number of active and inactive compounds (Figure 1). Of the 5-HT_1A_ ligands not used in the development of the models, the sets of active ligands were selected (i) randomly (marked in green in Figures 1, 4-6 and Figures S1–S6), (ii) to be the most diverse (blue), or (iii) in a way reflecting the abundance of different scaffolds according to manual clustering (red). Out of the 438 compounds from the ChEMBL database with *K*_i_ (5-HT_1A_) > 1000 nM, the 200 most diverse compounds (diversity-based selection tool in Canvas) constituted the set of decoys for the above-mentioned ligand groups (Figure 1). Similarly, the active ligands were complemented with compounds with assumed inactivity from the DrugBank database (low potential for binding to 5-HT_1A_ receptor was confirmed by SEA search tool [46]). The most diverse molecules with polarizable nitrogen and no data regarding activity toward the 5-HT_1A_ receptor were selected. All statistical parameters were calculated as an average of the values obtained for the pair of sets that was composed of the same actives (Figure 1). 

A validation set was created out of the novel 5-HT_1A_R ligands that appeared in the ChEMBL database version 10 (May 2011). The inactive compounds from this set were a challenge for the method because they were very similar to active compounds. Similarity search using MOLPRINT 2D fingerprint and Tanimoto metric revealed that 24.8% of inactives compounds had similarity coefficient with actives of 0.9 or higher.

### Search for the best linear combination of models

The process of selecting the optimal linear combination was conducted using an in-house script (see Figure S7) because the amount of data and number of combinations (hypotheses from three clustering methods, two hit modes and three different actives sets) rendered manual evaluation nearly impossible. The tool recursively generated all possible combinations of a given length and selected a top-scored combination in terms of the optimized parameter, namely, the Mathews Correlation Coefficient (MCC), accuracy or recall which was obtained using the average of the values received for the pairs of actives vs. the assumed inactives and the actives vs. the decoys.

$$MCC=\frac{TP\cdot TN-FP\cdot FN}{\sqrt{\left(TP+FP)(TP+FN)(TN+FP)(TN+FN\right)}}$$

$$Accuracy=\frac{TP+TN}{TP+FP+TN+FN}$$

Recall=TPTP+FN

Where *TP* stands for the number of true positives (actives labeled as actives), *TN* – true negatives, *FP* – false positives (inactives labeled as actives) and *FN* – false negatives.

MCC takes values from –1 to +1, where +1 represents perfect prediction, 0 represents random prediction and –1 represents an inverse prediction, whereas the accuracy and recall ranged from 0 to 1.

Two modes of compound filtering were evaluated. The “hit-once” mode classified the ligand as active if it was recognized by at least one of the models in the combination and in the “hit-twice” mode if at least two of the hypotheses flagged the ligand as active.

## Results

**Figure 7:**
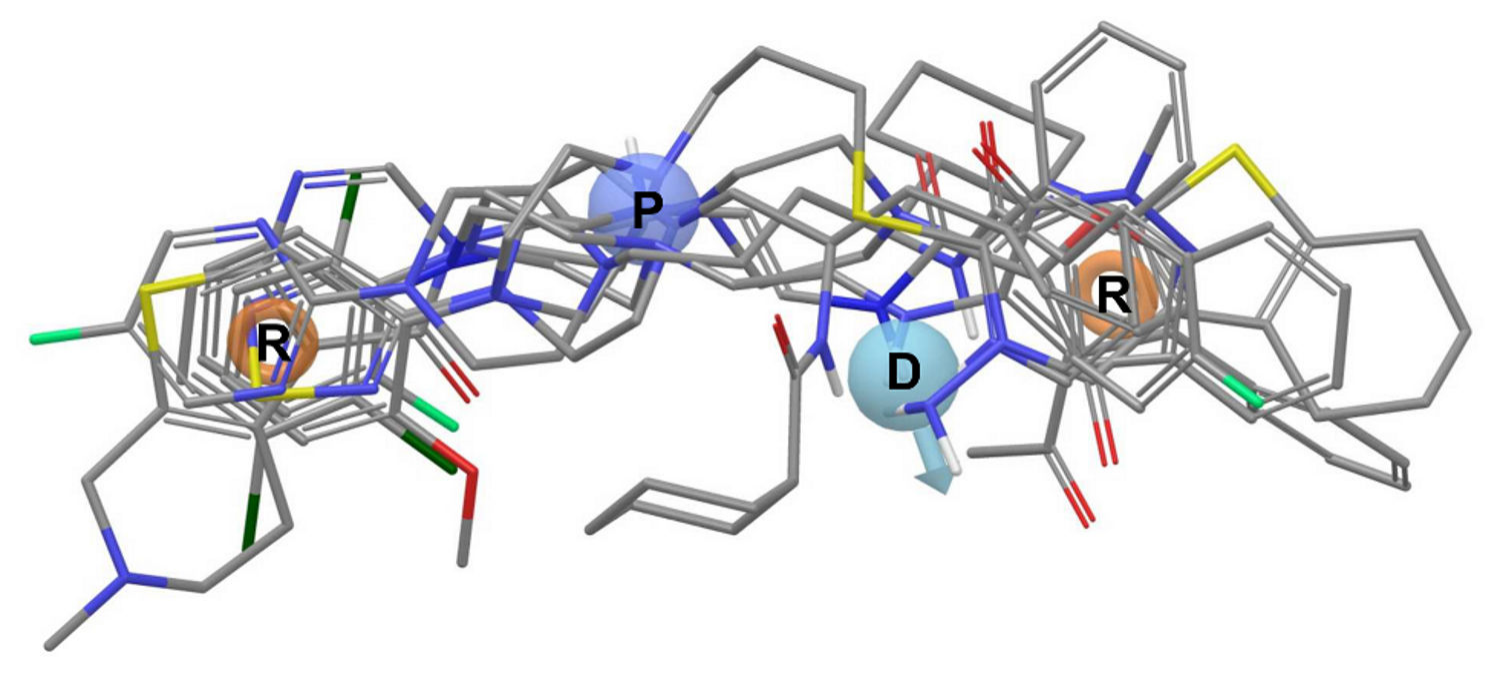
Exemplary pharmacophore hypothesis selected for arylpiperazines with classical amide fragment. Exemplary pharmacophore hypothesis selected for arylpiperazines with classical amide fragment mapping 6 out of 10 cluster representatives. The model fit 462 of the 533 compounds (87%) in the cluster. The feature abbreviations are: hydrogen-bond donor – **D**, positively charged group – **P**, aromatic ring – **R**.

Because the method is designed for VS, various factors influencing filtering performance were investigated. Starting from the active compounds clustered using three different methods, a series of pharmacophore hypotheses were developed (one model per cluster, see sample hypothesis in Figure 7). From the pool of singular models, linear combinations of various lengths were formed (the hypotheses retrieved for different clustering methods were not mixed) and evaluated using diverse test sets. Three coefficients were optimized at two restriction levels (hits must have been recognized by at least one or two models): MCC, accuracy and recall, as the standard measures of screening performance. 

### Development of the optimal linear combination

**Figure 8:**
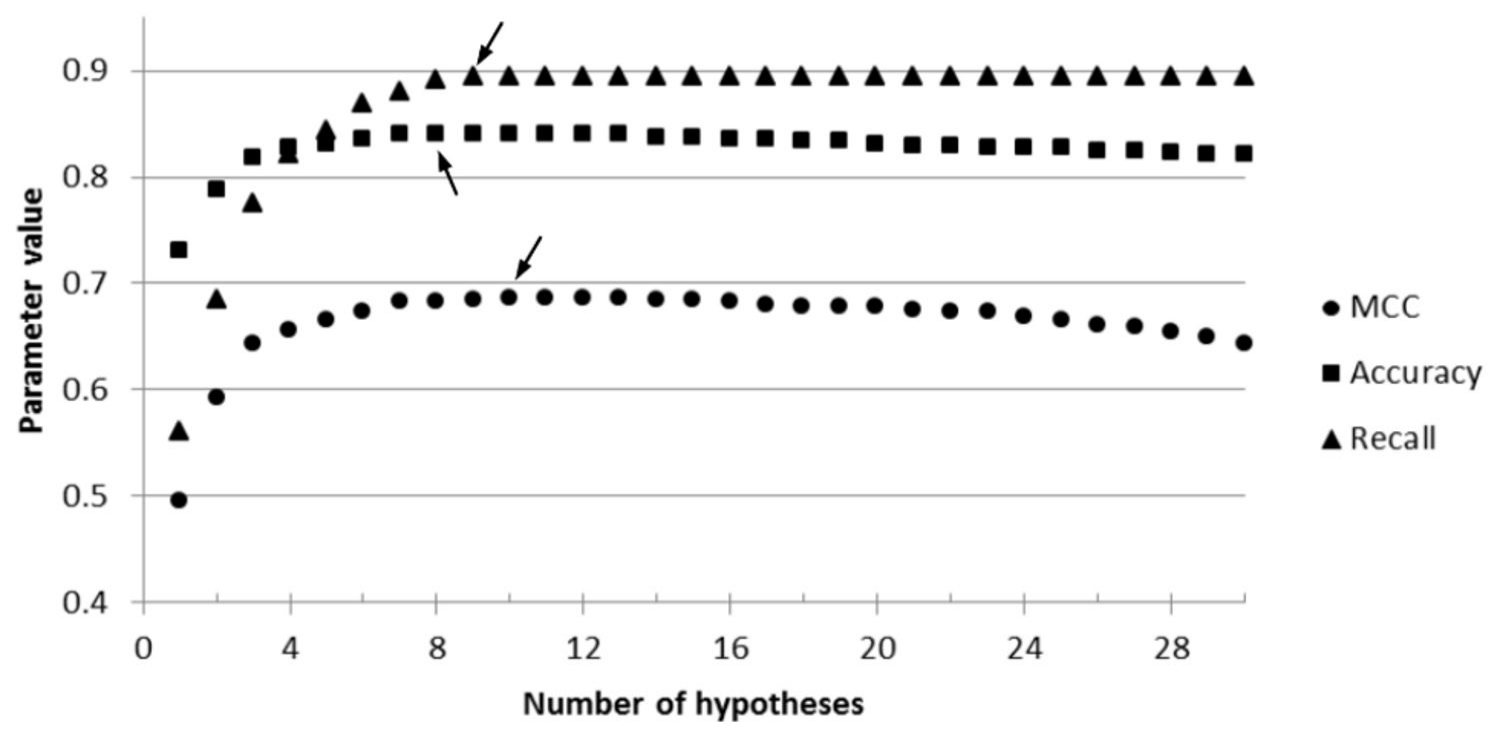
An optimization curve for the investigated parameters of a top-ranked linear combination of MCC. An optimization curve for the investigated parameters of a top-ranked linear combination of MCC (M2D/random/hit-once); arrows indicate the maximum value: MCC reached a rate of 0.686 for 10 hypotheses (also see Figure 6); the optimization of accuracy and recall had the highest values for a combination of 8 and 9 hypotheses, respectively (also see Figures S1 and S2).

The analysis of the approximation to the optimal ensemble of models showed that adding subsequent hypotheses allows for the saturation of the chemical space of the 5-HT_1A_R ligands until the maximum value of the optimized parameter is reached (Figure 8).

**Figure 9:**
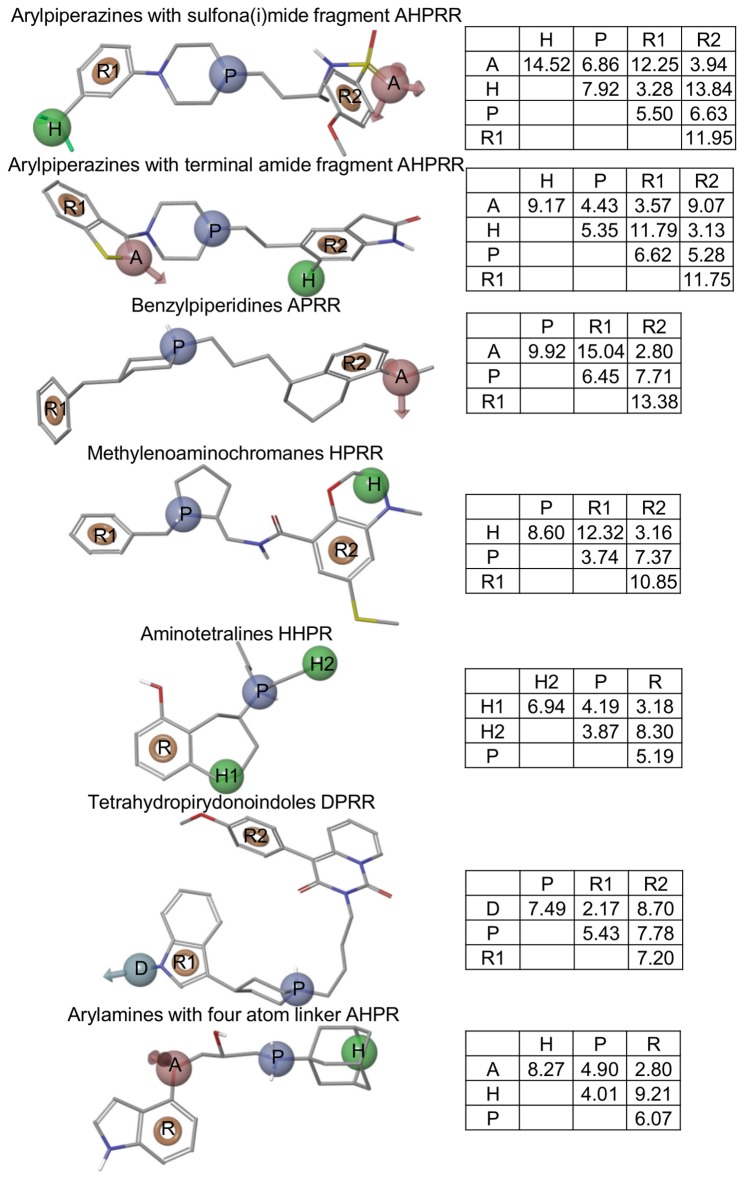
The best linear combination of pharmacophore models obtained for manual clustering and MCC optimization. The best linear combination of pharmacophore models obtained for manual clustering and MCC optimization (manual/random/hit-once; see also Figures 4 and 6). For each hypothesis the best fitting compound is presented, along with a matrix of distances (in angstroms) between features and a name of cluster it was developed on. The feature abbreviations used are: hydrogen bond acceptor – **A**, hydrogen bond donor – **D**, hydrophobic group – **H**, positively charged group – **P**, aromatic ring – **R**.

The maximization of the MCC parameter led to 6–11 models long combinations for the hit-once and 10–13 of those for the hit-twice mode, depending on the test set/clustering scheme, and the range of the maximum MCC values was from 0.427 to 0.686 (Figure 4). Figure 9 shows details of the MCC-optimized linear combination of 7 models developed on manual clustering, random test set and hit-once mode. The MCC at the highest level indicates misclassifications of only 12% of the active ligands and of one third of the inactive ligands. The experiments proved that the “hit-once” method was slightly better than the “hit-twice” method, and the difference between the best respective combinations was 0.069. In terms of clustering methods, the M2D and manual methods outmatched the approach based on 3D pharmacophore fingerprints.

The analysis of the top-scored combinations revealed frequent occurrences of short hypotheses (formed from four or five features), yet the size of the cluster, the feature count of the pharmacophore model and the pharmacophore efficiency could not be correlated with the performance of the combination. For example, the benzylpiperidines cluster (consisting of only 55 compounds) produced a short, four-feature hypothesis occurring in 17 of 18 optimized combinations. However, the hypothesis representing the largest cluster (other arylpiperazines) was not part of any combination. The results may also suggest that the hypotheses with high feature counts (e.g. ergolines with a seven-element hypothesis) are too strict to participate in optimal combinations; however, there is no statistically significant evidence to support this statement (Figures 5, S5, S6 and S8).

Regarding accuracy optimization, the process established the length of the combination on 6–11 hypotheses for the hit-once and 11–16 for the hit-twice approach. Again, the M2D fingerprint-based clustering method led to the best results (an accuracy of 0.840 for the random actives test set). The hit-once method of optimization was better than the hit-twice; however, this difference did not exceed 0.049. The details of the experiments can be found in Figure S1 .

The optimization of the recall returned compositions of 9 to 21 pharmacophore models for the hit-once method and 12 to 25 for the hit-twice method, and the values of the recall ranged from 0.445 to 0.920 (Figure S2 ). In that case, the combination curve reached a plateau after climbing to the maximum value. This effect was caused by a lack of FP count in the parameter definition; thus, the misclassifications were ignored. The results for the hit-once method were significantly better than those for the hit-twice selection. In addition, the combination for the hit-twice selection was longer (the greatest difference in length was for the manual/diverse scheme (13 hypotheses)). In this experiment, the hypotheses based on manual clustering dominated because they provided the best combination of the populated actives set and the random actives, as well as the best overall linear combination in terms of recall.

### Validation results

An ensemble of top combinations in terms of MCC for each construction scheme was tested using the validation set (Figure 6), again showing the superiority of M2D clustering-based models and the hit-once search approach. The best MCC coefficient was 0.294, which was a low but acceptable value (MCC is normalized in the range of –1 to 1) for such a demanding test set. A validation set consisted of an imbalanced amount of active compounds (1423) in relation to decoys (286).

The validation of the best accuracy-optimizing combinations confirmed the advantage of the M2D clustering method. Manual clustering showed better results than the P3D method but was unable to compete with the M2D method. The highest accuracy obtained for a validation set was 0.710 for the M2D/populated/hit-once scheme (Figure S3). 

Combinations reaching the highest recall values achieved up to 0.743 for the validation set for the M2D/diverse/hit-once scheme (Figure S4). Again, in this case, the M2D clustering-based models performed better than the other models. The P3D clustering method also showed the worst results in that system.

### Random combination

The aim of this benchmark was to determine whether the performance of the combination of hypotheses was not influenced by size alone. For the three schemes that generated the best combinations for the respective statistic parameter (M2D/random/hit-once for MCC and accuracy and M2D/populated/hit-once for recall), ten random collections of hypotheses containing the same number of elements as the optimized hypotheses (10 for MCC and recall and 8 for accuracy) were prepared, and the respective statistics were calculated and averaged. The results (Table 1) clearly showed the superiority of the optimized combination over the random hypotheses ensemble, especially in the case of MCC.

**Table 1:** A comparison between the optimized parameter values and those obtained for randomly selected combinations consisting of the same number of single hypotheses as optimized combinations.

**Parameter**	**Optimized**	**Random**	**SD**	**Gain^*a*^**
MCC	0.686	0.504	0.086	36.10%
Accuracy	0.840	0.726	0.044	15.66%
Recall	0.920	0.773	0.047	19.02%

^a^ Percent increase of the value of the optimized statistical parameters compared with random combinations

The random values from ten different random linear combinations are averaged.

### Single hypothesis benchmark

The benchmark against the single pharmacophore hypothesis was essential in comparing its performance with the proposed approach. To cover the full chemical space of the 5-HT_1A_R ligands, a representative from each cluster was selected (either a centroid or a random pick) to develop a single (universal) hypothesis that was then tested on the validation sets. 

The results (Table 2) showed that the single hypothesis performed similarly to the P3D-based combination of hypotheses in terms of MCC. However, for all other parameters and combination schemes, the single hypothesis was significantly outmatched.

**Table 2:** A comparison between a single hypothesis and a linear combination of pharmacophore models.

**Clustering approach**	**Selection method**	**Hypothesis composition**	**Actives**		**Decoys**		**MCC**		**Accuracy**		**Recall**	
			**TP**	**FN**	**TN**	**FP**	**universal**	**optimized**	**universal**	**optimized**	**universal**	**optimized**
P3D	centroid	APRR	459	964	250	36	0.162	0.158	0.415	0.474	0.323	0.424
	random	AAPR	456	967	241	45	0.134	0.158	0.408	0.474	0.32	0.424
M2D	centroid	AHPR	639	784	227	59	0.184	0.294	0.507	0.71	0.449	0.743
	random	APRR	442	981	248	38	0.148	0.294	0.404	0.71	0.311	0.743
manual	centroid	APRR	399	1024	251	35	0.136	0.227	0.38	0.548	0.28	0.665
	random	APRR	390	1033	252	34	0.134	0.227	0.376	0.548	0.274	0.665

## Discussion

Because the method was designed for VS purposes, the high efficiency of such an experiment was the primary concern. The results showed an increased performance in the linear combination of pharmacophore models in VS compared with the single hypothesis, as measured using the standard parameters of MCC, accuracy and recall. The screening evaluation of the method, however, precipitated observations that require further discussion. 

The process of finding the optimal linear combination of pharmacophore models is resource- and time-consuming. The combination of twelve element sets out of twenty-four hypotheses led to nearly three million possible combinations. A subsequent evaluation of all of these combinations required a significant amount of resources and thus was a challenging task even for a powerful workstation. However, once the optimal combination is selected, the screening process is conducted in an amount of time comparable to that required by a single hypothesis approach.

The ensemble of pharmacophore models shows a reasonable performance in declining the compounds assumed to be decoys (up to 198 out of 200 properly classified), yet the more challenging true decoy set remains an issue. None of the proposed combinations found all of the active compounds from the test sets. The reason for this is the presence of clusters that did not produce a pharmacophore hypothesis that was suitable for screening and thus did not support the coverage for the chemical subspace of the active compounds. Thus, the importance of the choice of the clustering method (being the fundament of single hypothesis development) and algorithm used should be adjusted to the goals of the screening. The P3D method provided the best filter for decoy structures, but its performance in finding active compounds was significantly weaker, thus lowering the measured VS parameters. However, M2D showed an increased rate of locating active compounds at the cost of decoy recognition. M2D is the best method given all optimized parameters. The performance of manual clustering appeared to be a balance between the aforementioned algorithms (the ratios of TP and TN were acceptable), and the parameter-measured performance of manual clustering was not drastically lower than that for M2D. The manual division of compounds can to some extent compete with automatic approaches; however, the time consumption and human factor impacting the final outcome provide disincentives to the wide use of manual clustering. Nevertheless, this splitting method provided structural information unavailable from different approaches, and moreover, reported the classification of entire chemical space of the 5-HT_1A_R ligands stored in ChEMBL.

The approach requiring the selection of one ligand using at least two hypotheses appeared to be too strict. The results proved that different hypotheses primarily do not overlap each other, leading to an increased number of false negatives in the VS experiments and thus significantly reduced screening parameter values.

## Conclusions

The results showed improved performance of the proposed method in virtual screening experiments. All investigated VS parameters outmatched both single hypothesis and random linear combination approaches. The experiments also proved that the automatic method of hierarchical clustering (based on the MOLPRINT 2D fingerprint) is a good option for screening. The computational cost of optimization increased, but the outcome compensated for that increase. Given the proposed method’s success, it will be incorporated into our screening workflow [9] and applied for the next extended set of targets. Further improvement of the script interface will be undertaken, thus making it usable for other research groups. 

## Supporting Information

Figure S1 **The optimized values of accuracy for each possible scheme.** Length of combination is shown on top of the bars.
(TIF)

Figure S2 **The optimized values of recall for each possible scheme.** Length of combination is shown on top of the bars.
(TIF)

Figure S3 **The accuracy results of the validation of top linear combinations.** Length of combination is shown on top of the bars.
(TIF)

Figure S4 **The recall results of the validation of top linear combinations.** Length of combination is shown on top of the bars.
(TIF)

Figure S5 **A composition of each top ranked linear combination, obtained for P3D clustering procedure.** The length row contains the total number of hypotheses forming a respective top ranked combination. Values of optimized statistical parameters for manual clustering are shown in 4 and for accuracy and recall in Figures S1 and S2, respectively.
(TIF)

Figure S6 **A composition of each top ranked linear combination, obtained for M2D clustering procedure.** The length row contains the total number of hypotheses forming a respective top ranked combination. Values of optimized statistical parameters for manual clustering are shown in Figure 4 and for accuracy and recall in Figures S1 and S2, respectively.
(TIF)

Figure S7 **Pseudocode of in-house script (about 300 lines) used for the search for the best linear combination.**
(PDF)

Figure S8 **Venn diagrams containing numbers of elements common for the same optimizing schema (clustering approach/actives test set/hit mode) for different screening parameters.** Number in overlapping area indicates elements present in two or more linear combinations. Number of hypotheses not used in any of the top-ranked combinations is beyond the circles. Numbers in circles sum up to the size of the given top combination (Figures 5, S5 and S6).
(TIF)
