# Supplementary figures and images for: A Linear Combination of Pharmacophore Hypotheses as a New Tool in Search of New Active Compounds – An Application for 5-HT1A Receptor Ligands
Source: PLoS One. 2013 Dec 18;8(12):e84510. doi: 10.1371/journal.pone.0084510 (PMC3867515; doi:10.1371/journal.pone.0084510)

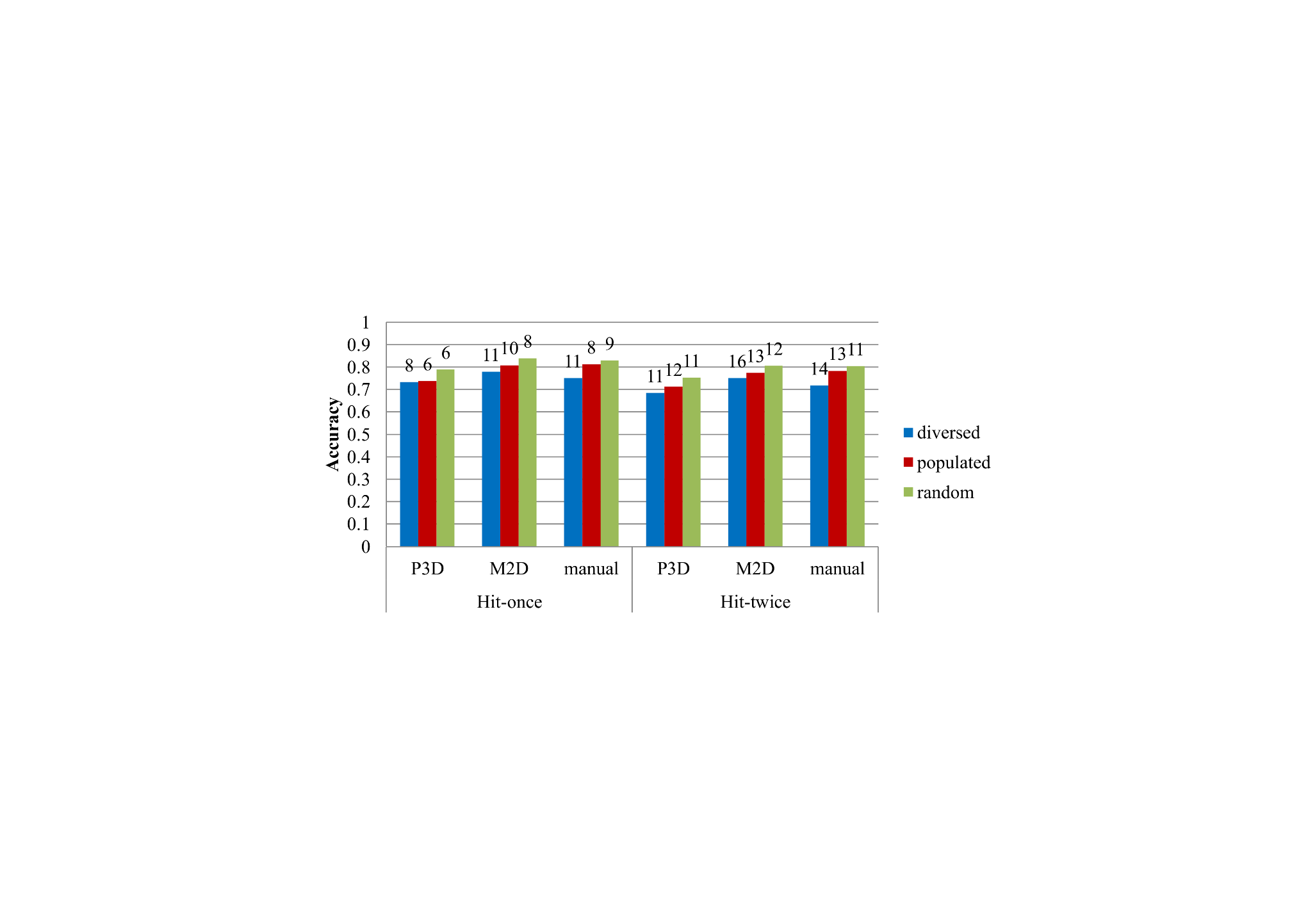

Supplement: Figure S1 — The optimized values of accuracy for each possible scheme. Length of combination is shown on top of the bars. (TIF) [file pone.0084510.s001.tif]

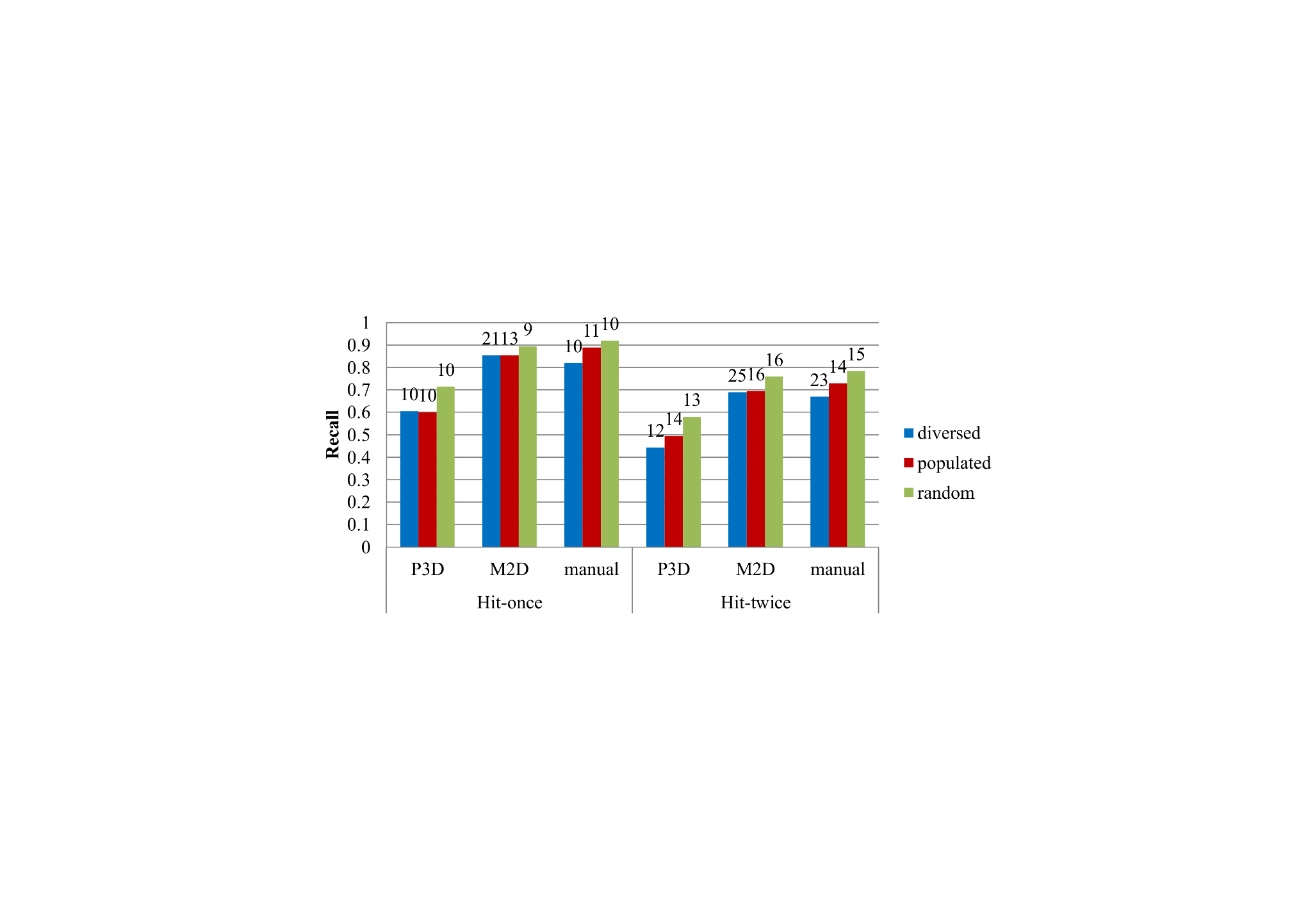

Supplement: Figure S2 — The optimized values of recall for each possible scheme. Length of combination is shown on top of the bars. (TIF) [file pone.0084510.s002.tif]

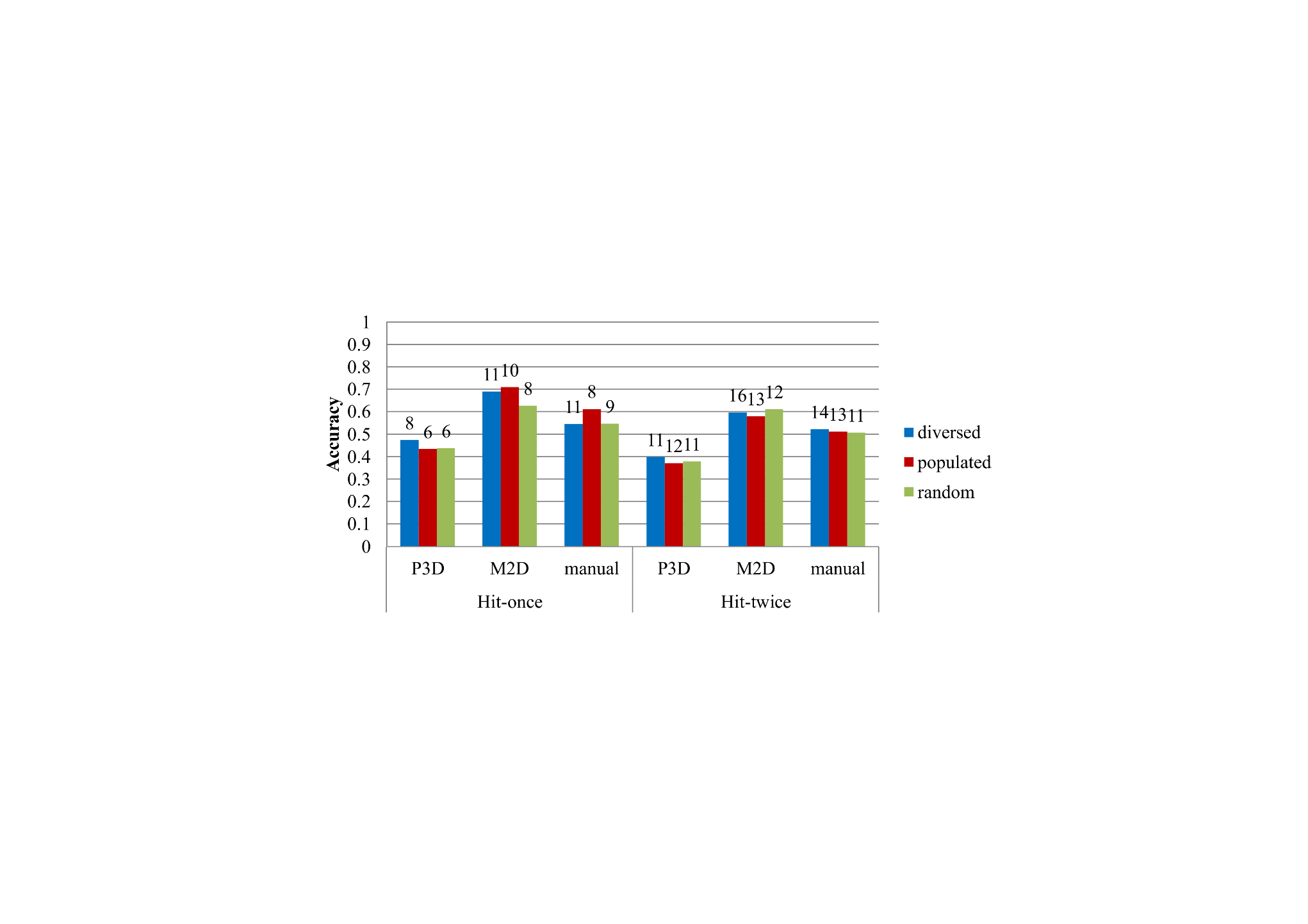

Supplement: Figure S3 — The accuracy results of the validation of top linear combinations. Length of combination is shown on top of the bars. (TIF) [file pone.0084510.s003.tif]

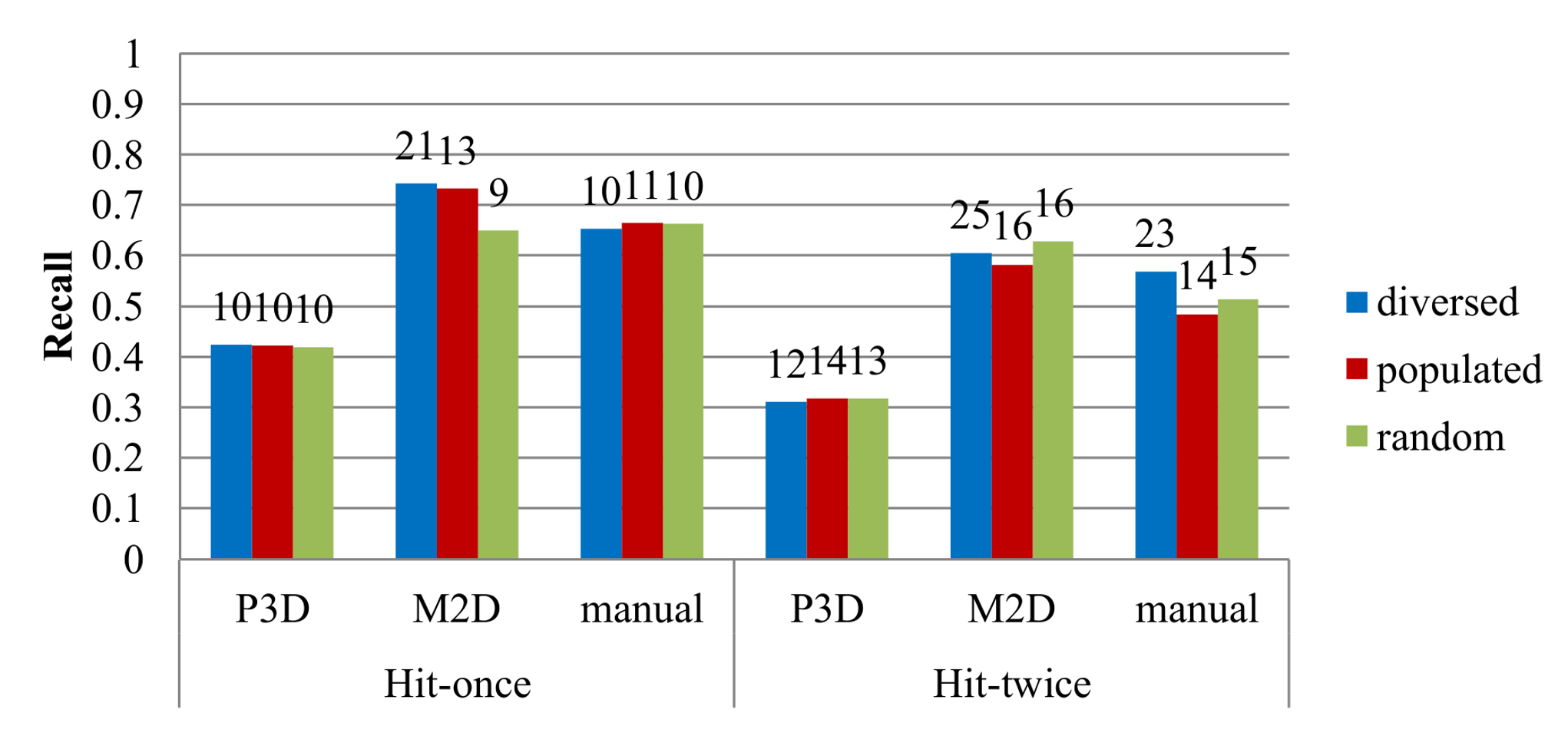

Supplement: Figure S4 — The recall results of the validation of top linear combinations. Length of combination is shown on top of the bars. (TIF) [file pone.0084510.s004.tif]

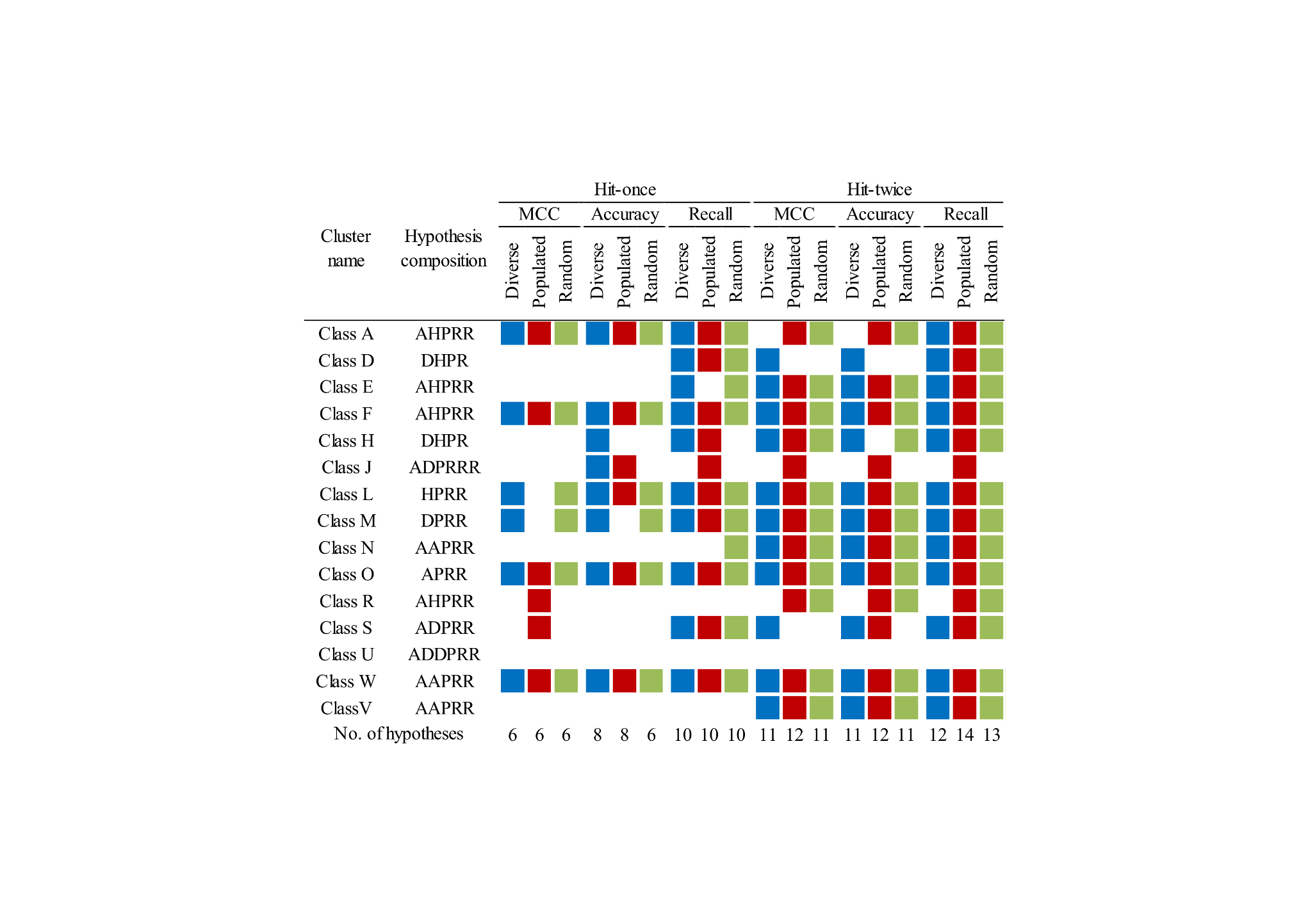

Supplement: Figure S5 — A composition of each top ranked linear combination, obtained for P3D clustering procedure. The length row contains the total number of hypotheses forming a respective top ranked combination. Values of optimized statistical parameters for manual clustering are shown in 4 and for accuracy and recall in Figures S1 and S2, respectively. (TIF) [file pone.0084510.s005.tif]

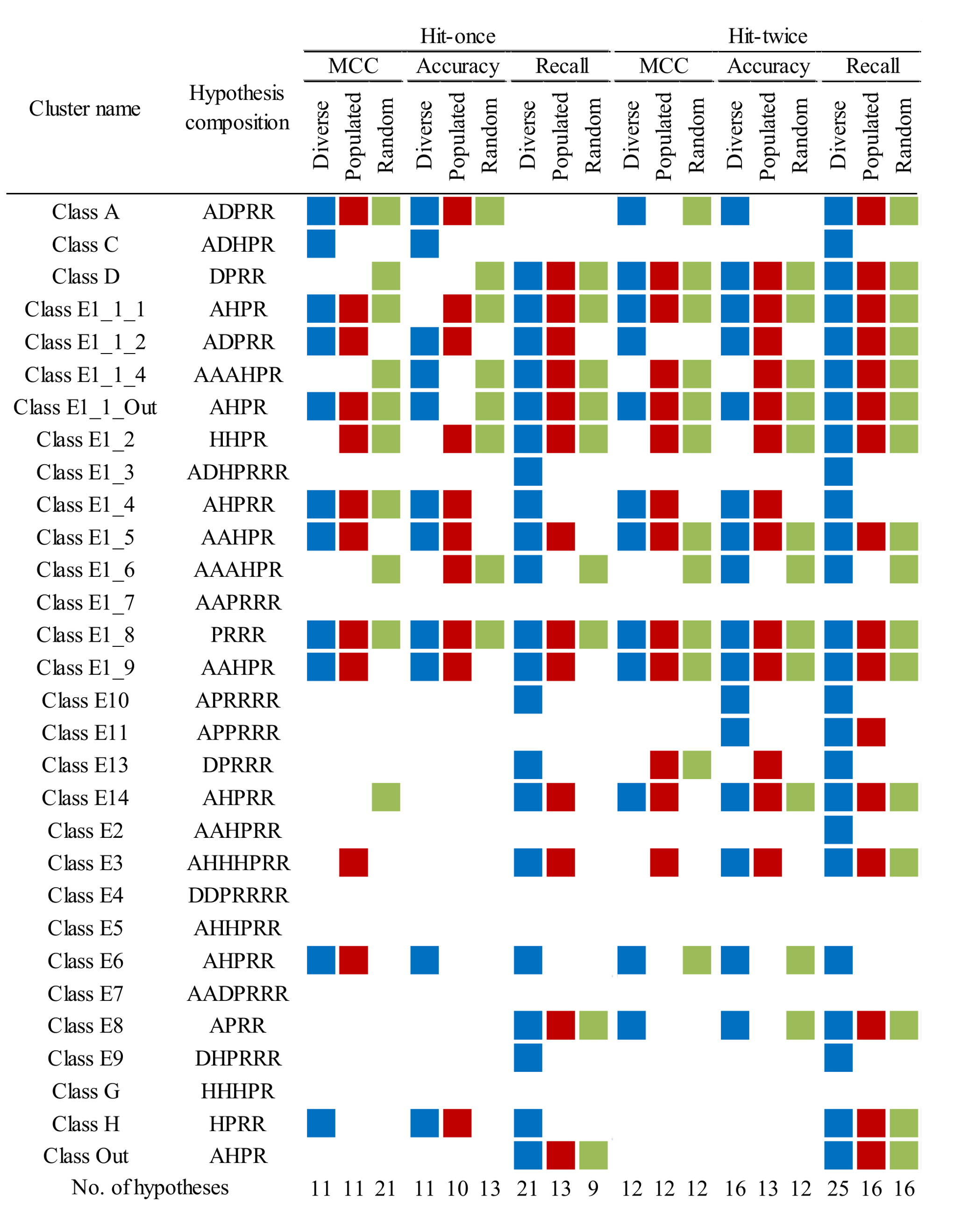

Supplement: Figure S6 — A composition of each top ranked linear combination, obtained for M2D clustering procedure. The length row contains the total number of hypotheses forming a respective top ranked combination. Values of optimized statistical parameters for manual clustering are shown in Figure 4 and for accuracy and recall in Figures S1 and S2, respectively. (TIF) [file pone.0084510.s006.tif]
