# Supplementary material for: A Linear Combination of Pharmacophore Hypotheses as a New Tool in Search of New Active Compounds – An Application for 5-HT1A Receptor Ligands
Source: PLoS One. 2013 Dec 18;8(12):e84510. doi: 10.1371/journal.pone.0084510 (PMC3867515; doi:10.1371/journal.pone.0084510)
Supplement: Figure S7 — Pseudocode of in-house script (about 300 lines) used for the search for the best linear combination. (PDF) [file pone.0084510.s007.pdf]

Choose an appropriate statistical parameter (MCC, accuracy, recall)
Choose an appropriate clustering approach (P3D, M2D, manual)
Choose an appropriate active test set (diverse, populated, random)
Choose an appropriate hit mode (hit-once, hit-twice)
j= number of elements in linear combination
for j=1, …, n do
    (i) Generate all possible linear combinations
    (ii) Calculate selected statistic parameter for each combination
    (iii) Select maximum value (max.j)
        if max.j > max.j–1
            compute max.j+1
        else break
end for
Output: Optimized value of selected statistics, composition of optimal combination and number of TP, TN, FP, FN.
